# Sympatric and allopatric niche shift of endemic *Gypsophila (Caryophyllaceae)* taxa in the Iberian Peninsula

**DOI:** 10.1371/journal.pone.0206043

**Published:** 2018-11-07

**Authors:** Miguel de Luis, Carmen Bartolomé, Óscar García Cardo, Juan Manuel Martínez Labarga, Julio Álvarez-Jiménez

**Affiliations:** 1 Departamento de Ciencias de la Vida, Facultad de Ciencias, Universidad de Alcalá, Alcalá de Henares (Madrid), Spain; 2 Empresa Pública de Gestión Ambiental de Castilla-La Mancha (GEACAM), Cuenca, Spain; 3 Departamento de Sistemas y Recursos Naturales, E.T.S.I. Montes, Forestal y del Medio Natural, Universidad Politécnica de Madrid, Madrid, Spain; Oklahoma State University, UNITED STATES

## Abstract

Several species of the *Gypsophila* genus are endemic to the Iberian Peninsula, including gypsophytes of particular ecological, evolutionary and biochemical interest, and taxa that have undergone both sympatric and allopatric genetic differentiation. The niche shift among these taxa has been assessed using ecological niche modelling and ordination techniques, adopting a niche overlap approach to compare the similarity and equivalency of the ecological niches. We used the Maximum Entropy method to study the potential distribution of these taxa in different eras: the Last Glacial Maximum (LGM), the Mid Holocene and the current conditions. We present evidence of niche shift during the speciation of *G*. *bermejoi*, with a strong niche overlap between the parental taxa (*G*. *struthium* subsp. *struthium* and *G*. *tomentosa*), yet both overlap much more weakly with the hybrid species. This phenomenon may be explained by genetic and epigenetic interactions, and it has been described in other species. We also studied the sister subspecies *G*. *struthium* subsp. *struthium* and *G*. *struthium* subsp. *hispanica*, with mostly allopatric distributions and with the Iberian System mountain range acting as a geographical barrier. The Iberian System and other mountain ranges may have favored differences in the climatic conditions on either side of the mountain range, which is consistent with an incipient process of bioclimatic ecological speciation. These results seem to indicate that niche shift can occur over very different timespans. In the case of *G*. *bermejoi*, speciation may have produced significant niche shifting in one or two generations due to its alloploid nature. By contrast, *G*. *struthium* subsp. *struthium* and *G*. *struthium* subsp. *hispanica* seem to have undergone a more gradual process of allopatric genetic differentiation driven by bioclimatic factors. Both these processes are relatively recent and they will have been strongly influenced by the climate change at the end of LGM.

## 1-Introduction

**Fig 1:**
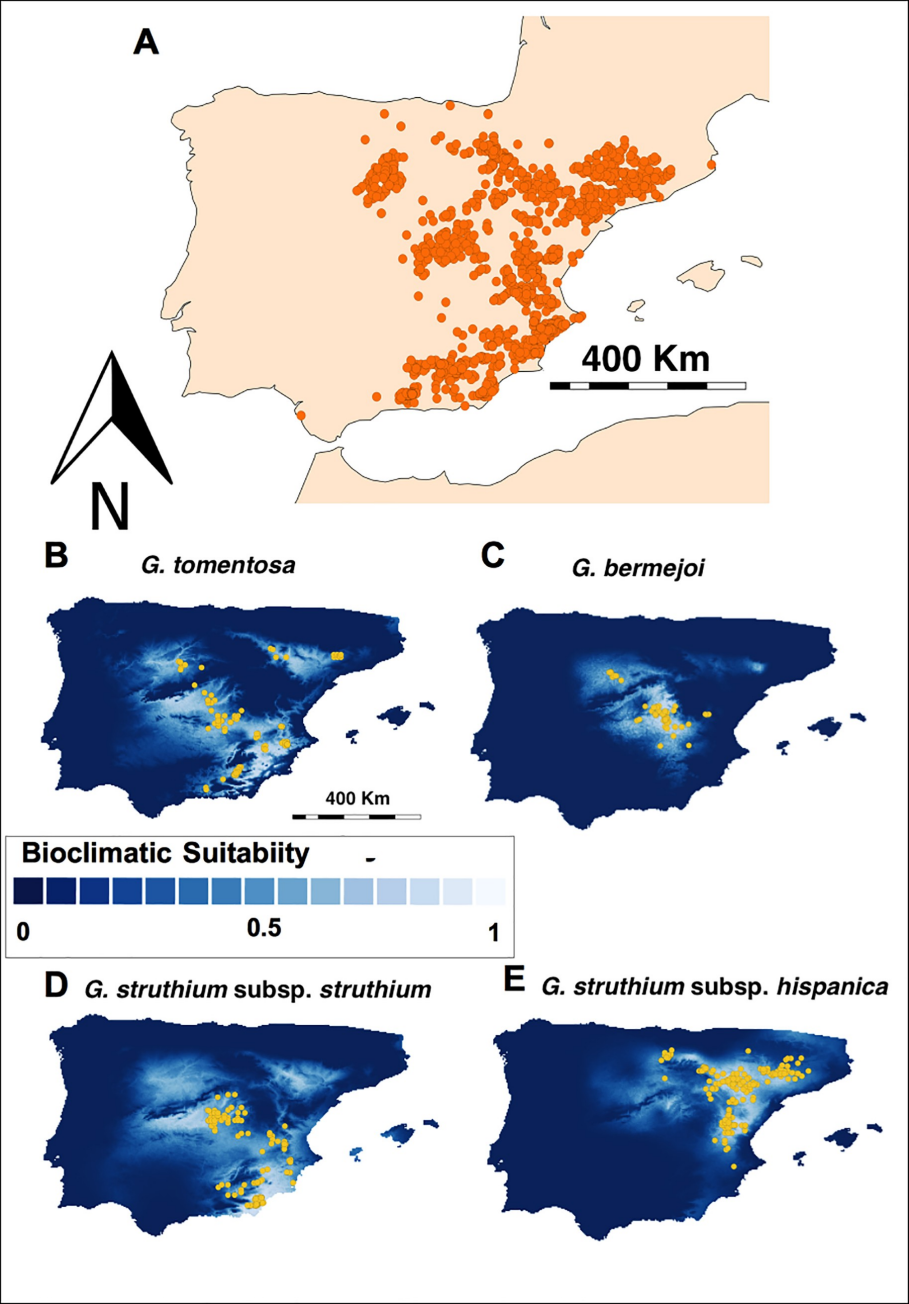
(A) Map of the Iberian Peninsula showing the presence of *Ononis tridentata* L., a plant that is indicative of gypsum and gypsiferous loam soils. (B to E) MaxEnt maps showing the bioclimatic suitability for *G*. *tomentosa* (B), *G*. *bermejoi* (C), *G*. *struthium* subsp. *struthium* (D) for *G*. *struthium* subsp. *hispanica* (E). We have included the points where the taxon is currently present (yellow dots).

Gypsum outcrops in the Iberian Peninsula constitute challenging and unusual habitats for plants. Considered to be the only reservoir of these soils in Europe [1], they are ranked as priority areas for conservation by the European Union [2]. These habitats are scattered in patches, concentrated in the eastern half of the Peninsula (Fig 1A), a fragmented distribution that is not dissimilar to the biogeography of island ecosystems [1]. Although gypsum soils are challenging environments for plants, they harbor a diversity of flora that includes many endemic species. Thus, they are of particular interest to study plant evolution and ecology.

The *Gypsophila* L. genus (Caryophyllaceae) has its origins in the Caucasus Mountains and the Irano-Turanian region [3–6], with its center of diversity in Turkey [7], although it now includes some Iberian endemisms. In the Iberian Peninsula this genus contains 9 taxa included in 8 species (López González, 1990), of which 5 are endemics for Spain: *G*. *montserratii* Fernández Casas, *G*. *struthium* subsp *strutihum* L., *G*. *struthium* subsp *hispanica* (Willk.) G. López, *G*. *bermejoi* G. López, *G*. *tomentosa* L. [8]. The last four can be considered as gypsophytes according to the expert criteria [9,10] (Fig 1B to 1E). The species of *Gypsophila* are typical of rock-dwelling habitats or in situations of little edaphic development and **l**ithosols (*G*. *repens* L., *G*. *montserratii*, *G*. *struthium*), or alteration habitats such as roadsides, disturbed land and farmlands (*G*. *bermejoi*, *G*. *tomentosa*, *G*. *elegans* MB, *G*. *muralis* L. and *G*. *pilosa* Hudson); almost all species can characterize steppes.

Gypsiferous soils represent difficult environments for plants, most remarkably for their strong xericity, the presence of a crusty surface and serious nutritional imbalances [11]. Gypsophytes are of interest in biochemical terms due to their tendency to accumulate certain minerals and their ability to produce specific secondary metabolites. As such, the production of antioxidants, like phenols and flavonoids, and that of saponins has been studied in *Gypsophila* [12–14]. Moreover, the singular ecological conditions of these soils has produced plants highly adapted to their environment, with an abundance of endemic species. This circumstance, along with the fragmentation of their habitats, makes these species a suitable model to study plant evolution and speciation [15].

While the origin of the genus has been established in the Neogene period, c.a. 5.7 Million years ago (Mya: [16]), it remains unclear when gypsophytes became established in the Iberian Peninsula. Given that the Messinian Salinity Crisis [17,18] ended at the start of the Pliocene, it is possible that these plants could have arrived on the Iberian Peninsula at that time and had their current distributions affected by Quaternary climate changes [19]. We set out to assess the role of these climatic oscillations in the speciation and niche shift of the Iberian *Gypsophila* endemisms, as well as the timespan of these effects. Indeed, these taxa offer instances of both sympatric and allopatric genetic differentiation.

In terms of the Iberian *Gypsophila* species, here we have studied two subspecies of *Gypsophila struthium* L. that are mostly found in allopatric ranges [20]. In terms of their distribution, *G*. *struthium* subsp. *hispanica* is mainly found in the northeastern quadrant of the Iberian Peninsula, whereas *G*. *struthium* subsp. *struthium* occupies the southeast quadrant. Both zones are separated by the Iberian System mountain range. Some studies have been carried out into the structure and genetic diversity of these two subspecies [21], revealing weak but significant genetic differentiation between them, possibly due to a more recent diversification in the Pliocene (5.3–2.6 Mya) and Pleistocene (2 Mya-10,000 years ago) epochs [22]. In this interval of approximately 5.3 Mya to about 10,000 years ago, the climate is known to have undergone notable changes that have had a decisive influence on the genetic structure of populations of different organisms [21].

Species Distribution Models show that these climatic oscillations have forced significant changes in the range of these taxa [23]. These changes, along with the existence of the Iberian System mountain range, which serves as a boundary between the distribution zones of *G*. *struthium* subsp. *struthium* and *G*. *struthium* subsp. *hispanica* (Fig 1D and 1E), may have had an important role in favoring different climatic conditions between the ranges of both taxa. Indeed, these climatic changes may perhaps have been as important as the physical barrier that blocked the genetic flow between both subspecies, since a zone of contact does exist between them. Bayesian analysis reveals populations with a mixture of ancestral gene clusters where both subspecies overlap [22]. Both these characteristics indicate that the reproductive barrier existing between these subspecies **i**s incomplete, which we consider to be similar to cases of allopatric speciation that may be currently in progress. The fact that the differences in the bioclimatic conditions on both sides of the Iberian System seem to have influenced the genetic differentiation between both subspecies would make this situation similar to a case of ecological speciation. Ecological speciation can be defined as the emergence of barriers between populations due to divergent selection based on ecological factors. That is, natural selection would arise as a consequence of the interaction of organisms with their environment or with other organisms [24]. According to our data, climate seems to have played a key role in this divergent selection. However, the influence of other factors cannot be ruled out, such as biotic factors, the interaction of biotic and climatic factors, or phenomena such as genetic drift or a founder effect.

We have also considered a tetraploid allopolyploid gypsophyte from an ecological point of view, *Gypsophila bermejoi* G. López [25]. Its parental species are *G*. *struthium* subsp. *struthium* [25] and *G*. *tomentosa* [25]. Polyploidy is characterized by the presence of two or more sets of paired or homologous chromosomes in each nucleus, in this case both sets of chromosomes proceeding from different species (Allopolyploidy). This phenomenon is responsible for the appearance of new species and it is a crucial event in the diversification of species, particularly in plants [26]. This process requires a few generations to become established and we wanted to determine if it may be associated with an ecological niche shift. In general, one parental genome is preferentially expressed over the other in both natural and synthetic allopolyploids [27]. In these species, new phenotypes may arise through genetic and epigenetic phenomena, and they generally occupy environments that differ from those occupied by their progenitors. Furthermore, the polyploid hybrids that form may express extreme traits [28–32]. Thus, it makes sense to think that this phenomenon will affect the ecological niche of the hybrid species.

Species Distribution Models have been employed successfully to study a wide range of ecological situations [33–36]. These tools can help understand the effect of quaternary climatic oscillations and the possible changes in the range of species over time. Such models could highlight whether climate change had a significant influence on the speciation process that gave rise to *G*. *bermejoi* [23], and on the genetic differentiation between the other *G*. *struthium* subspecies. Accordingly, we have used ecological niche tests to assess the possible niche shift in these taxa. The data obtained point to a central role of bioclimatic changes on the evolution of *Gypsophila* in the Iberian Peninsula.

## 2-Materials and methods

### 2.1-Study area and species

The area studied here includes the gypsum steppes of the Iberian Peninsula, a territory with an approximate extent of 623,000 km^2^ located between latitudes 36°00’08” N—43°47’38” N and longitudes 9°29’00” O—3°19’00” E. The gypsum outcrops in this territory cover a total extension of 32,487 km^2^ (6.1% of the Peninsula) [1], and they are concentrated in the eastern half of the Peninsula under the influence of a Mediterranean climate.

In this study, we focused on the genetic differentiation of distinct taxa of *Gypsophila* (*Caryophyllaceae)*: *Gypsophila bermejoi* (2n = 68), an allopolyploid species found on verges and slopes in areas of gypsum soil; its parental species, *G*. *struthium* subsp. *struthium* (2n = 34) and *G*. *tomentosa* (2n = 34); and *G*. *struthium* subsp. *hispanica* (2n = 34), a sibling taxon of *G*. *struthium* subsp. *struthium*. All these perennial plants are endemic to the Iberian Peninsula, inhabiting salt-rich substrates and fundamentally, gypsum soils.

### 2.2-Data sources

Two types of data are required for the modeling of ecological niches using MaxEnt and for niche comparisons: species occurrence data and a set of environmental data for those locations. Here, we used species occurrence data from the Global Biodiversity Information Facility (GBIF) database [37]. These data were carefully filtered. For instance, we detected some wrong citations of *G*. *struthium* subsp. *hispanica* in the range of *G*. *struthium* subsp. *struthium*. In terms of the environmental variables, we used the bioclimatic variables from The WorldClim portal (www.worldclim.org) that makes publically available data for 19 bioclimatic variables [38]. These data are presented as a set of world maps for the different bioclimatic variables, which are the extrapolated output of values taken from meteorological stations distributed around the world. In this same platform, past and future maps of these bioclimatic variables can also be found, generated using different climate models. In this study we also used the bioclimatic maps for the mid-Holocene and the Last Glacial Maximum (LGM), generated by the Community Climate System Model (CCSM4). This model unifies four separate models of the atmosphere, land, oceans and sea-ice, generating a simulation of the global climate [39]. In this study, we only used selected bioclimatic variables according to the ecological profile of the species studied and the climatic limiting factors of the area (Table 1). Moreover, as we only considered variables with r <0.95 [40], the number of localities for some species is relatively low due to the endemic nature of the taxa. The number of variables selected, seven in this case, must be lower than 10 x the number of species occurrences in order to prevent over-parameterization in the niche modeling techniques used [41,42].

**Table 1:** List of the environmental variables selected to implement both the MaxEnt species distribution models and the models for niche comparison.

Variable	Source	Resolution
bio1—Annual Mean Temperature	WorldClim	2.5’
bio4—Temperature Seasonality	WorldClim	2.5’
bio6 –Minimum Temperature in the Coldest Month	WorldClim	2.5’
bio7– Temperature Annual Range	WorldClim	2.5’
bio12 –Annual Precipitation	WorldClim	2.5’
bio15 –Precipitation Seasonality	WorldClim	2.5’
bio17 –Precipitation of the Driest Quarter	WorldClim	2.5’

### 2.3-Maximum Entropy (MaxEnt) Model

To model ecological niches and areas of potential distribution, we used the Maximum Entropy (MaxEnt) approach [43,44]. The algorithms required are currently implemented in R language and using a graphic interface (freely available at http://biodiversityinformatics.amnh.org/open_source/maxent/). For this work the most recent version of the program available was used (MaxEnt 3.4.1: for an explication of the method, see [43–47]). Basically, this tool assesses the probability of the presence of the species in function of the environmental variables selected. The inputs are data files on the presence of the species and a series of maps of the environmental variables considered to be significant for initial values that can be interpreted as an index of suitability of the habitat for that particular species [43]. We dealt with the autocorrelation issues by eliminating the redundant presence in each pixel on the scale of the bioclimatic variables used. We used 70% of the presence records to train the models and 30% to test them.

Two statistics were used to assess the models’ predictive performances: the Area Under the Curve (AUC) and True Skill Statistics (TSS). For the AUC approach, we have to plot the Receiver Operating Characteristic (ROC) curve. This curve represents sensitivity (True Positive Rate) vs. 1- specificity (True Negative Rate) at various threshold values. Or in other words, this curve is the true positive rate as a function of the false positive rate. Thus, If we obtain a diagonal line our model is uninformative (not better than random guessing). Increasing area under the ROC curve implies better model fit [43]. TSS takes into account both omission and commission errors, and success as a result of random guessing. It does express the hit rate relative to the false positive rate. TSS values ranges from -1 to +1 (perfect agreement). Values of zero or less indicate a performance no better than random [48].

This study focused on the effect of climatic oscillations and as such, we used bioclimatic variables to generate the models. Given the strict gypsophilic nature of these plants, the real suitability is 0 in all the locations without gypsum soils. To trace the areas with gypsum soils we chose to use records of the presence of a gypsum indicator plant, *Ononis tridentata L*. [10,11]. Then we obtained the bioclimatic suitability values only for those points, for the case of *G*. *bermejoi* and its parental species. For *G*. *struthium* subsp. *struthium* and *G*. *struthium* subsp. *hispanica*, these suitability values were taken at the current presence points of the taxon and those of its sister subspecies. All these taxa are confined to gypsum soils but, in the second case, our goal was to asses the suitability of each subspecies in its sister’s range.

In choosing the functions “feature”, we deactivated the functions hinge and threshold. In this way, the response curves obtained were easier to interpret and they were better adjusted to the ecological theory of the niches [47].

### 2.4-Niche comparison

The different niches of the *Gypsophila* taxa were compared using the method developed by Broennimann *et al*. (2012) [48,49], an approach used successfully in a wide range of plant, animal and virus case studies [50–56]. The method adopts an ordination approach to assess the occurrence density along two environmental axes. The bioclimatic variables included are reduced using a Principal Component Analysis (PCA) correlation matrix [57] and the environmental space is defined by the first two axes of the PCA, which is limited by the minimum and maximum environmental values across the whole study area. The environmental space is divided into a grid of *r* x *r* cells, where each cell corresponds to a unique combination of environmental conditions. The species occurrences are plotted onto this environmental space grid and a kernel smoothing method is then applied to them. We have plotted the niches of the species in pairs to make their comparison easier [58].

Niche overlap is calculated using Schoener’s D metric [59], a metric that ranges from 0 (no overlap) to 1 (complete overlap) [60]. In this approach, an unbiased estimate of D is calculated using a kernel density function that is applied to the occurrence densities. Statistical significant niche overlaps are then determined using niche similarity and niche equivalency tests [49,60], the latter determining whether the niches of two taxa are equivalent in two ranges. In other words, whether the niche overlap is constant when randomly redistributing the occurrences of both taxa among the two geographical areas. All these occurrences are pooled and randomly relocated into two datasets, maintaining the number of occurrences in the original datasets. This was repeated 100 times to generate a null distribution that could be compared with the D metric observed. It is important to note that non-significant results only mean that the niches are not identical, not that they are dissimilar. The niche similarity test determines whether the overlap between niches in two ranges is different from the overlap between the observed niche in one range and niches selected at random from the other range. The random niches are generated by randomly shifting the centroid of the second taxon in the environmental space. Again, the process is repeated 100 times to generate a null distribution. Niche similarity tells us if one taxon’s niche is better at predicting the second groups niche than randomly generated niches from the background area.

## 3-Results

From the models generated with MaxEnt for the three studied species, the AUC values obtained were 0.965 (*G*. *bermejoi*), 0.934 (*G*. *tomentosa*), 0.933 (*G*. *struthium struthium*) and 0.936 (*G*. *struthium hispanica*), all of which were sufficiently high to accept these models. The TSS values for the same species were 0.833 (*G*. *bermejoi*), 0.686 (*G*. *tomentosa*), 0.621 (*G*. *struthium struthium*) and 0.691 (*G*. *struthium hispanica*), with values above 0.6 considered to be good and 0.2–0.6 to be fair to moderate.

### 3.1-Range changes in Gypsophila since Last Glacial Maximum (LGM)

The climatic oscillations during the Quaternary may have had an important influence on the evolution of the taxa studied. To assess the possible range changes, we used MaxEnt to project these models onto the climatic conditions calculated for the Last Glacial Maximum (LGM), the Mid-Holocene period (MH) and the present. The maps we obtained showed important changes in the potential distribution of *G*. *struthium* subsp. *struthium* and *G*. *struthium* subsp. *hispanica*.

**Fig 2:**
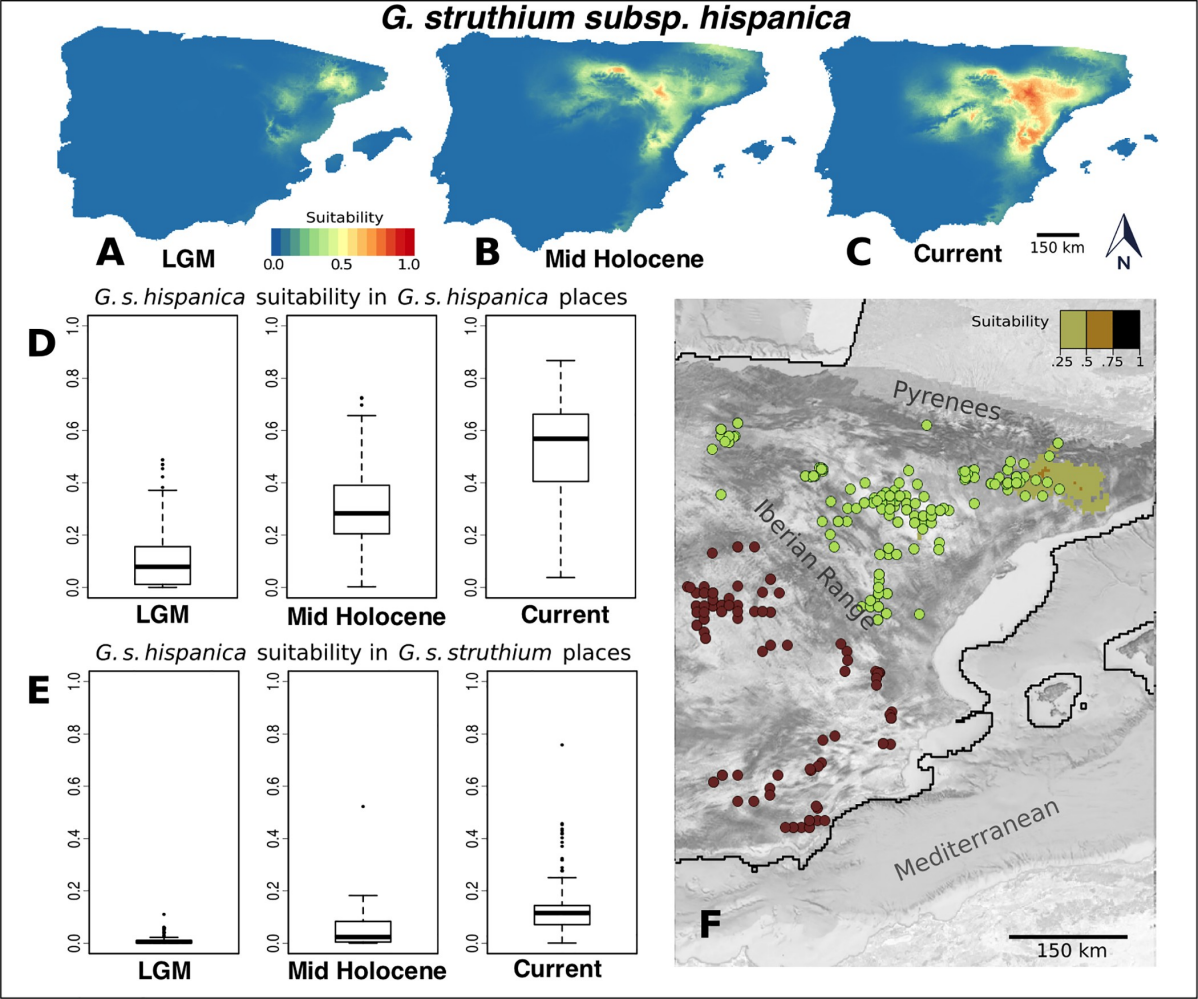
Habitat suitability predicted by the MaxEnt models for *G*. *struthium* subsp. *hispanica*. Habitat suitability map for the Last Glacial Maximum (A), the Mid-Holocene period (B) and in the current climatic conditions (C). (D) Habitat suitability values (R software standard boxplot) in the Last Glacial Maximum, the Mid-Holocene and in the current climatic conditions measured at the points where the taxon is currently present. In (E) we show a similar plot but the values of suitability for *G*. *struthium* subsp. *hispanica* are taken at the current locations of its sister subspecies, *G*. *struthium* subsp. *struthium*. (F) Current distribution of *G*. *struthium* subsp. *struthium* (brown dots) and *G*. *struthium* subsp. *hispanica* (light green dots) over the Glacial Maximum suitability map for *G*. *s*. *hispanica* (values > 0.25). Imagery: PNOA 2018 CC-BY 4.0 ign.es.

For *G*. *struthium* subsp. *hispanica* the maximum suitability areas during the LGM are not far from its current distribution. These areas lie south of the eastern Pyrenees, in the Ebro valley and in a region close to the eastern coast line of the Iberian Peninsula. The current distribution for this taxon is shifted slightly towards the west, to areas where there are patches of gypsum soils that make it possible that this taxon existed there during the LGM. Nevertheless, the values of climatic suitability in gypsum soil areas are very low (Fig 2A).

**Fig 3:**
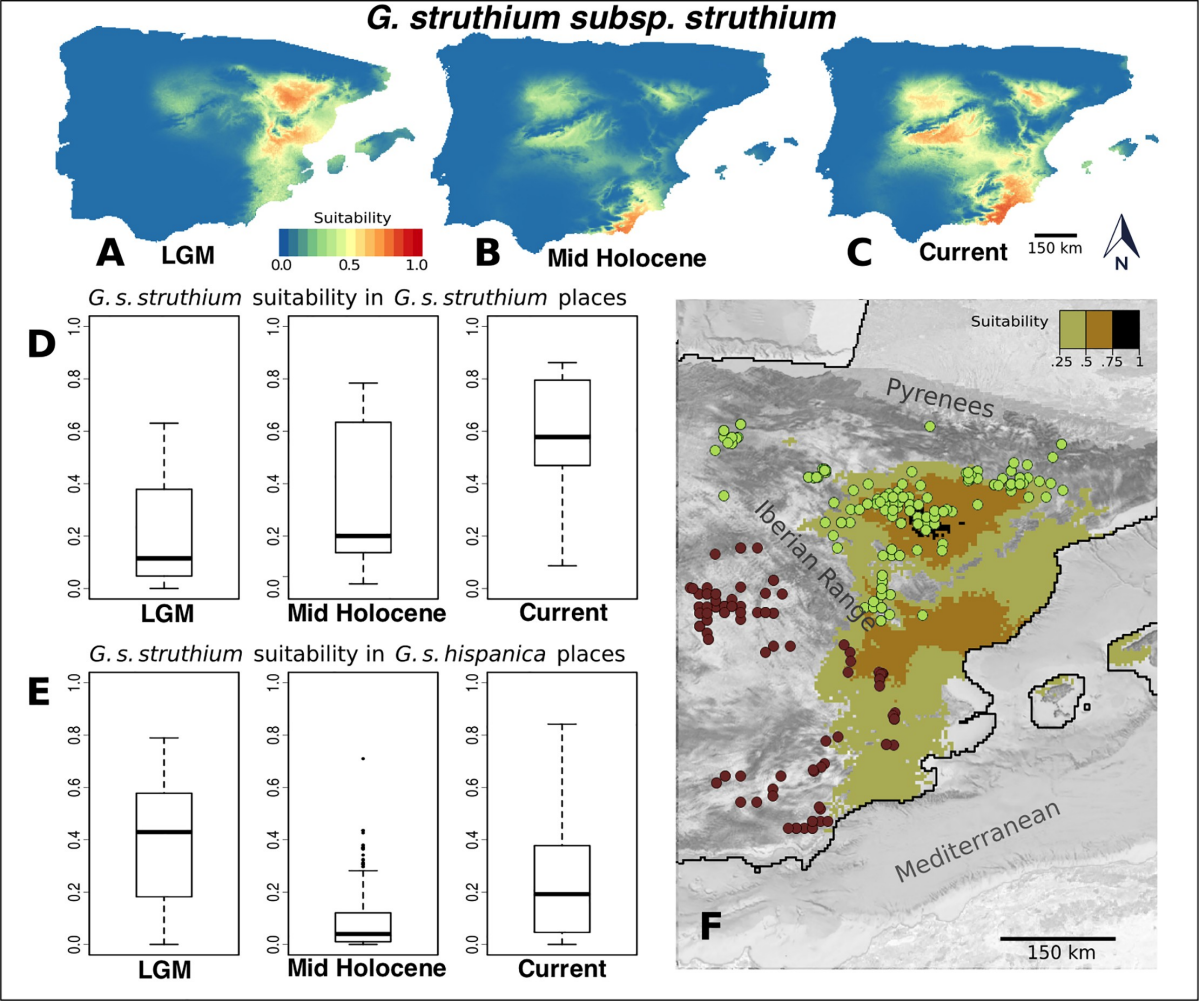
Habitat suitability predicted by the MaxEnt models for *G*. *struthium* subsp. *struthium*. Habitat suitability map for the Last Glacial Maximum (A), the Mid-Holocene period (B) and in the current climatic conditions (C). (D) Habitat suitability values (R software standard boxplot) in the Last Glacial Maximum, the Mid-Holocene and in the current climatic conditions measured at the points where the taxon is currently present. In (E) we show a similar plot but the values of suitability for *G*. *struthium* subsp. *struthium* are taken at the current locations of its sister subspecies, *G*. *struthium* subsp. *hispanica*. (F) Current distribution of *G*. *struthium* subsp. *struthium* (brown dots) and *G*. *struthium* subsp. *hispanica* (light green dots) over the Glacial Maximum suitability map for *G*. *s*. *struthium* (values > 0.25). Imagery: PNOA 2018 CC-BY 4.0 ign.es.

By contrast, the climatic suitability for *G*. *struthium* subsp. *struthium* during the LGM appeared to be strong in the Ebro valley and in the eastern coastal region. These areas overlap with the maximum suitability in that period for *G*. *struthium* subsp. *hispanica* but the suitability for *G*. *struthium* subsp. *struthium* at those areas seemed to be much higher (Fig 3A and 3E).

We also obtained the suitability values from the MaxEnt models at the current locations for both subspecies during the LGM, the Mid-Holocene period and in the current climatic conditions. As expected, the suitability values predicted by the model of *G*. *struthium* subsp. *hispanica* at its current location increase over time (Fig 2D). Moreover, the suitability values for this subspecies at the present points of *G*. *struthium* subsp. *struthium* never reach high values. As a general rule, yet with some exceptions (see outliers in Fig 2E), *G*. *struthium* subsp. *hispanica* never performed very well in the current range of *G*. *struthium* subsp. *struthium*. By contrast, the maps for *G*. *struthium* subsp. *struthium* reflected a quite different behavior, whereby the suitability of this subspecies measured at its current sites increased gradually from the LGM (Fig 3). We observed a different trend for the values measured in present conditions for the sister subspecies. At the current sites of *G*. *struthium* subsp. *hispanica*, the *G*. *struthium* subsp. *struthium* was well suited in the LGM and it seems to have been very comfortable in the Ebro valley, currently an important domain in the range of *G*. *struthium* subsp. *hispanica*. Thus, the climatic transition to the Mid Holocene would appear to have turned this region into a much less favorable area for *G*. *struthium* subsp. *struthium*.

**Fig 4:**
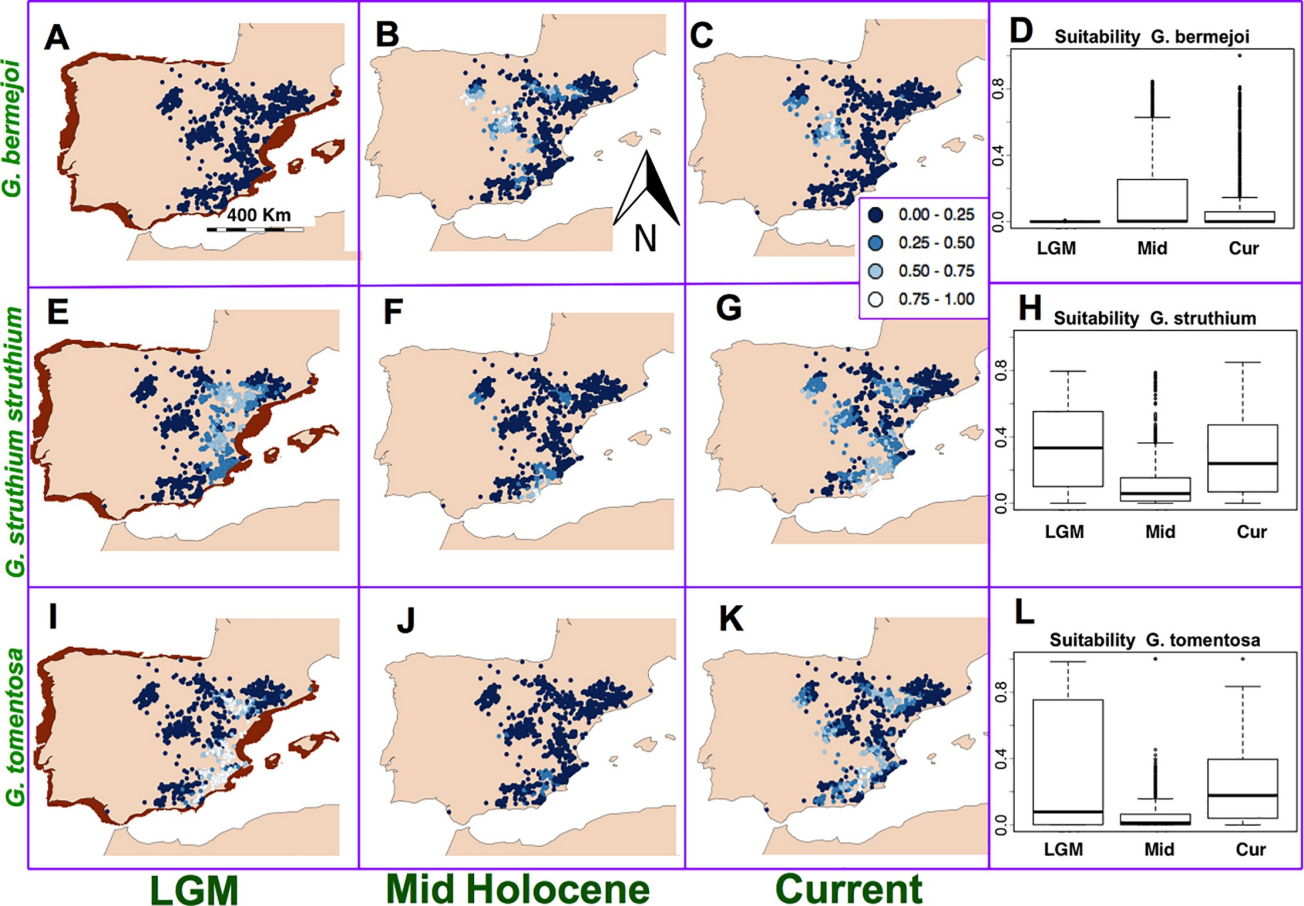
Habitat suitability predicted by the MaxEnt models for *G*. *bermejoi* and its parents for the locations with suitable soils. Due to the gypsophilic character of these species the real suitability is zero in soils other than gypsum and gypsiferous loam soils. Habitat suitability map for *G*. *bermejoi* during the Last Glacial Maximum (A) (notice the different coast lines for this period), the Mid-Holocene period (B) and in the current climatic conditions (C). (D) Habitat suitability values (R software standard boxplot) in the Last Glacial Maximum, the Mid-Holocene and in the current climatic conditions measured at the locations with gypsum soils. Notice that A and the first plot in D show an extremely low suitability for *G*. *bermejoi* during the LGM. It is very likely that the speciation event was effective only after the end of LGM. The panels series E to H and I to L, show similar information relative to *G*. *struthium* subsp. *struthium* and *G*. *tomentosa* (parental species of *G*. *bermejoi*) respectively. The parental species show a high degree of sympatry during the LGM and probably they could interbreed. Even though, it is likely that the climatic conditions were preventing the hybrid *G*. *bermejoi* to thrive during that climatic period.

To study the case of *G*. *bermejoi* we generated maps for the bioclimatic suitability values taken only in locations with favorable soils for this species and its parental taxa (Fig 4). Again, the models show meaningful changes in the potential distribution of *G*. *bermejoi*, *G*. *struthium* subsp. *struthium* and *G*. *tomentosa*. (Fig 2).

During the LGM both, *G*. *struthium* subsp. *struthium* and *G*. *tomentosa* seemed to take shelter on the eastern and south eastern coastal strip of the Iberian Peninsula, with this zone extending into inland locations along the Ebro Valley (Fig 4E and 4I). They show a high degree of sympatry. In contrast, the suitability for *G*. *bermejoi* is almost zero during this period (Fig 4A). This can be seen more precisely in the box plots (Fig 4D, 4H and 4L).

This situation changes remarkably during the Mid Holocene. In most of the gypsum outcrops, bioclimatic suitability decreases for *G*. *struthium* subsp. *struthium* and *G*. *tomentosa*. But in the case of *G*. *bermejoi* this climatic transition seems to favor its presence in many locations of the Iberian Peninsula. The bioclimatic suitability values for this species increases markedly for this period.

The current climatic conditions, again, favor higher bioclimatic suitability values in most of the favorable locations for the parental taxa (Fig 4G and 4K). The evolution of these values through can be clearly seen in Fig 4H and 4L. *G*. *bermejoi* shows a very different behavior. For the current climatic conditions its bioclimatic suitability decreases. But now, the box plots show a relatively high number of outliers (Fig 4D). These values correspond to the limited number locations where this plant feels comfortable, as expected in an endemic species.

### 3.2-Comparison of ecological niches

**Fig 5:**
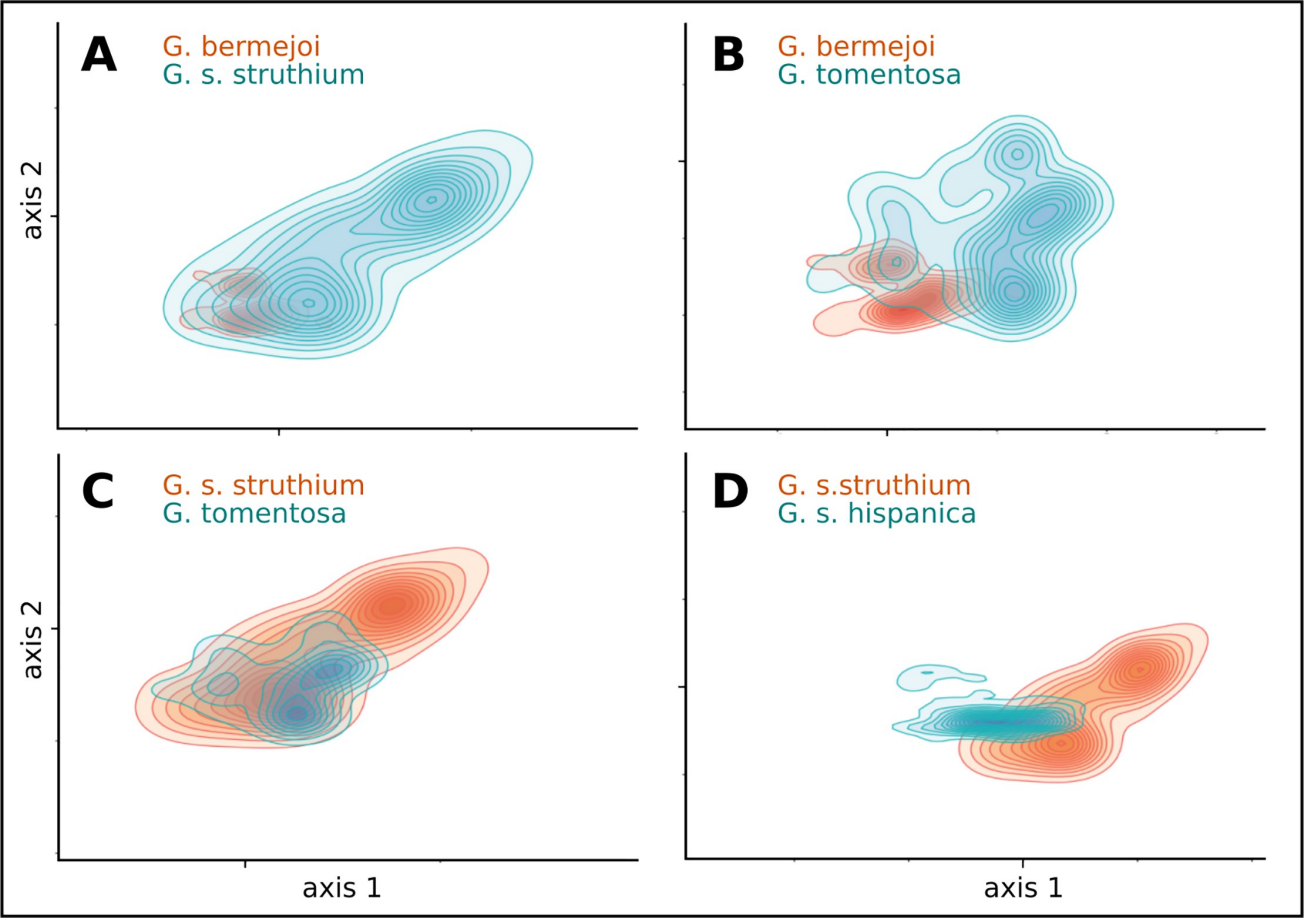
PCA plots for the *Gypsophila* taxa studied. The environmental space is defined by the first two axes of the PCA analysis and it is limited by the minimum and maximum environmental values across the whole study area. Contour lines draw suitability values [58]. The different taxa have been plotted in pairs to make comparisons easier: (A) *G*. *bermejoi* (red) vs. *G*. *struthium* subs. *struthium* (blue), (B) *G*. *bermejoi* (red) vs. *G tomentosa* (blue), (C) *G*. *struthium* subs. *struthium* (red) vs. *G*. *tomentosa* (blue), (D) *G*. *struthium* subs. *struthium* (red) vs. *G struthium* subsp. *hispanica* (blue).

We compared the niches of these taxa using niche equivalence and niche similarity tests (Table 2). In the case of the sympatric speciation of *G*. *bermejoi*, we compared its ecological niche to those of its parental species, *G*. *struthium* subsp. *struthium* and *G*. *tomentosa*. In the first case the results show that the ecological niches are neither equivalent nor similar. In the second one, there could be some degree of niche similarity but not equivalency. The values of Schoener’s D between the parental species (*G*. *struthium* subsp. *struthium* and *G*. *tomentosa)* are relatively high and this is of no surprise given that the plants must coincide to some degree to be able to interbreed. Interestingly, the values of niche overlap are much lower when these parental species are compared to the hybrid species *G*. *bermejoi*. These data, along with the PCA (Fig 5) plots favors the existence of niche shift during the formation of *G*. *bermejoi*.

**Table 2:** Schoener’s D and niche similarity tests for Iberian *Gypsophila* taxa. The first number in the cells corresponds to the observed value of Schoener’s D. The not significant P-values are indicated by “ns”.

TAXA		NICHE SIMILARITY		NICHE EQUIVALENCY
a	b	a -> b	b -> a	
***G*. *struthium struthium***	***G*. *struthium hispanica***	**0.1723** P-value = 0.2475 **ns**	**0.1723** P-value = 0.2772 **ns**	**0.1723** P-value = 0.0099 ***Not Equivalent***
	***G*. *tomentosa***	**0.4194** P-value = 0.0990 **ns**	**0.4194** P-value = 0.0396 **Similar**	**0.4285** P-value = 0.0099 ***Not Equivalent***
	***G*. *bermejoi***	**0.2015** P-value = 0.0891 **ns**	**0.2015** P-value = 0.0891 **ns**	**0.2015** P-value = 0.0099 ***Not Equivalent***
***G*. *tomentosa***	***G*. *bermejoi***	**0.2215** P-value = 0.0495 **Similar**	**0.2215** P-value = 0.0693 **ns**	**0.2215** P-value = 0.0099 ***Not Equivalent***

The PCA plots for *G*. *bermejoi* and its two parental species showed remarkable differences between the niches (Fig 5A and 5B), with *G*. *bermejoi* revealing itself to be a species with an ecological niche much narrower than those of *G*. *struthium* subsp. *struthium* and *G*. *tomentosa*.

For the sister allopatric subspecies of *G*. *struthium*, the results again showed that the niches are neither equivalent nor similar. Despite they being considered subspecies, the Schoener’s D value was comparable to those obtained between different species of this genus (Table 2). Moreover, the PCA plot again showed clear differences for the climatic niches of both taxa, with *G struthium* subsp. *hispanica* displaying a narrower ecological niche than *G*. *struthium* subsp. *struthium*.

## 4-Discussion

In this study, we have examined the evolution of several *Gypsophila* species endemic to the Iberian Peninsula, using similarity and equivalence tests to demonstrate evidence of niche shift in these species. The ecological niche of *G*. *bermejoi* is neither equivalent nor similar to that of its parental species, and a similar situation arises when the niches of the sister subspecies of *G*. *struthium* are compared [25].

The ecological niche of *G*. *bermejoi* has lower Schoener’s D values relative to both parental species, although higher values were obtained when comparing the niches of the parental species (*G*. *struthium* subsp. *struthium* and *G*. *tomentosa*). Indeed, the hybrid species that was derived from these parental types has neither an equivalent nor a similar niche. It is not uncommon for allopolyploid species to express extreme phenotypes and the phenotypic traits related to the ecological behavior of these species are no exception. Such a phenomenon would explain sudden ecological niche shift in one or two generations, a very short time span in evolutionary terms. A number of research works point to rapid epigenetic changes in allopolyploids genesis, under both synthetic and natural conditions **[31]**. According to Rieseberg **[30]**, large and rapid adaptive transitions can be explained by hybridization because “unlike mutation, it provides genetic variation at hundreds or thousands of genes *in a single generation*” (emphasis ours). Differences in gene expression have been observed between the diploid parental species and a synthetic F1 hybrid. According to Ainouche and Wendell **[28]** these results “showed that a significant fraction of expression bias found in allotetraploids likely *is initiated by genome merger per se*” (emphasis ours). We aim to demonstrate that the timespan for niche shift can vary widely. We think that speciation by hybridization could be in the fastest extreme of the timespan range.

There is evidence that climate is a crucial driving force in this event and Species Distribution Models indicate a sympatric distribution of the parental species during the LGM. The eastern coastal region was a refuge for both taxa, permitting the cross pollination between two species as occurred recently to produce the hybrid *Gypsophila x castellana* Pau [25]. However, environmental conditions prevented the hybrid from thriving due to its narrow bioclimatic niche and as such, climate would have repressed the speciation process throughout the Last Glacial period. Nevertheless, the climatic transition to the Holocene period would have triggered the formation of *G*. *bermejoi* [23].

By contrast, the formation of the *G*. *struthium* sister subspecies seems to have taken longer and it involves allopatry, although again the climate seems to be a driving factor in the genetic divergence of *G*. *struthium* subsp. *struthium* and *G*. *struthium* subsp. *hispanica*. The quaternary climatic oscillations produced critical changes in the ranges of the ancestral populations and the potential distribution of *G*. *struthium* subsp. *struthium* became limited to an eastern coastal strip of territory and the Ebro Valley during the LGM. The models produce high suitability values for this subspecies in these areas (Fig 3E), with a weaker habitat suitability signal for *G*. *struthium* subsp. *hispanica* over the same period, partially overlapping with the range of *G*. *struthium* subsp. *struthium*. Along with the absence of geographical barriers, this makes it difficult to accept the existence of *G*. *struthium* subsp. *hispanica* as a differentiated taxon during the LGM. Indeed, we favor the presence of ancestral populations similar to *G*. *struthium* subsp. *struthium* during this period.

The climatic change at the end of the Glacial Period enabled these populations to colonize or re-colonize the gypsum outcrops in the interior of the Iberian Peninsula. According to the MaxEnt maps, the climatic conditions during the Mid Holocene period favored the presence of *G*. *struthium* subsp. *struthium* in the central and south eastern regions of the Peninsula (Southwest and South of the Iberian System). Conversely, the suitability of this subspecies in the Ebro Valley (Northeast of the Iberian System) decreases abruptly. The climatic contrast between both areas was probably enhanced by the presence of the Iberian System mountain range [61,62], which makes it even more plausible that such climate changes exerted selective pressure on these populations. The outcome of this situation would be the genetic differentiation of the populations on either side of the mountains, similar to a process of ecological speciation [63–66].

We believe that most of the genetic differentiation between these subspecies occurred after the LGM. The models seem to point to the “in situ” substitution of *G*. *struthium* subsp. *struthium* by *G*. *struthium* subsp. *hispanica* in the Ebro Valley, and that climate was an important driving factor in this process. We favor a process similar to ecological speciation, in which the ancestral populations (similar to those of *G*. *struthium* subsp. *struthium*) are gradually replaced with *G*. *struthium* subsp. *hispanica* in the Ebro Valley. This scenario would explain why the current locations of *G*. *struthium* subsp. *hispanica* match almost perfectly with a high suitability region for *G*. *struthium* subsp. *struthium* during the LGM.

We want to stress that the complex genetic structure of both subspecies is not easy to explain and that the influence of other factors, both biotic and abiotic, cannot be ruled out. Nonetheless, the scenario could outline the recent evolutionary history of *G*. *struthium* subsp. *hispanica* and *G*. *struthium* subsp. *struthium*.

## 5-Conclusions

From the results obtained, we can draw the following conclusions:

- Different mechanisms for niche shift arise in the *Gypsophila* genus, affecting both sympatric and allopatric populations. In the former, new allopolyploid species can display a very different ecological behavior from the parental species. By contrast, in allopatric populations processes of genetic divergence may have been favored by the presence of a geographical barrier and different climatic conditions.
- The timespan of these processes can vary widely. Sudden niche shift was possible in the allopolyploid species *G*. *bermejoi*, with the new hybrid species likely appearing in one or two generations. For the separation of the two subspecies of *G*. *struthium*, our results point to a more gradual process of niche shift resulting from genetic differentiation that began at the end of the Last Glacial Maximum.
- It seems reasonable to discard the presence of *G*. *bermejoi* in the Iberian Peninsula during the LGM. This conclusion is supported by the fact of its very narrow bioclimatic niche (Fig 5A and 5B) Due to the sympatric distribution of its parental taxa during the LGM, we think that these taxa could have been interbreeding during that period, but the climatic conditions were repressing the speciation process. During the Mid Holocene the situation changes dramatically (Fig 4A and 4B) and now it is possible for the hybrid taxon (*G*. *bermejoi*) to thrive. We think that the effective formation of populations of *G*. *bermejoi* was posible only after the climatic transition between the LGM and the Mid Holocene took place [23].
- We favor a more ancestral character of *G*. *struthium* subsp. *struthium*. During the LGM, *G*. *struthium* subsp. *struthium* did it better in the current range of *G*. *struthium* subsp *hispanica*. Even better than in its current range (Fig 3D and 3E). During the same period, the situation is very different for *G*. *struthium* subsp. *hispanica*. Its suitability is almost 0 in the range of *G*. *struthium* subsp. *struthium* and poor in its own current range. This makes more difficult to accept the presence of *G*. *struthium* subsp. *hispanica* as a separated taxon in the Iberian Peninsula during this period (LGM). In others words: during the LGM *G*. *struthium* subsp. *struthium* seemed to feel at home in the locations where we now find the *G*. *struthium* subsp. *hispanica*. That is why we think that the ancestral populations of *G*. *struthium* subsp. *hispanica* in the Ebro Valley were more similar to the current *G*. *struthium* subsp. *struthium* and that they gradually evolved *in situ* to *G*. *struthium* subsp. *hispanica*.
- The quaternary climatic oscillations played a crucial role in this divergence and we believe it is impossible to disentangle the evolutionary history of these taxa without taking into account the quaternary climatic oscillations that occurred in the last 25,000 years.
- Despite the differences in mechanisms and timespan, several niche shift events in Iberian endemic *Gypsophila* taxa are relatively recent and they do not predate the LGM.

## Supporting information

S1 Table Habitat suitability predicted by the MaxEnt models for *G*. *struthium* subsp. *hispanica*, during the Last Glacial Maximum, the Mid-Holocene and in the current climatic conditions measured at the points where the taxon is currently present. (CSV)

S2 Table As S1 but the values of suitability for *G*. *struthium* subsp. *hispanica* are taken at the current locations of its sister subspecies, *G*. *struthium* subsp. *struthium*. (CSV)

S3 Table Habitat suitability predicted by the MaxEnt models for *G*. *struthium* subsp. *struthium*, during the Last Glacial Maximum, the Mid-Holocene and in the current climatic conditions measured at the points where the taxon is currently present. (CSV)

S4 Table As S3 but the values of suitability for *G*. *struthium* subsp. *struthium* are taken at the current locations of its sister subspecies, *G*. *struthium* subsp. *hispanica*. (CSV)

S1 Fig Niche similarity test histograms. (TIF)

S2 Fig Niche equivalency test histograms. (TIF)
