# Supplementary figures and images for: Sympatric and allopatric niche shift of endemic Gypsophila (Caryophyllaceae) taxa in the Iberian Peninsula
Source: PLoS One. 2018 Nov 7;13(11):e0206043. doi: 10.1371/journal.pone.0206043 (PMC6221283; doi:10.1371/journal.pone.0206043)

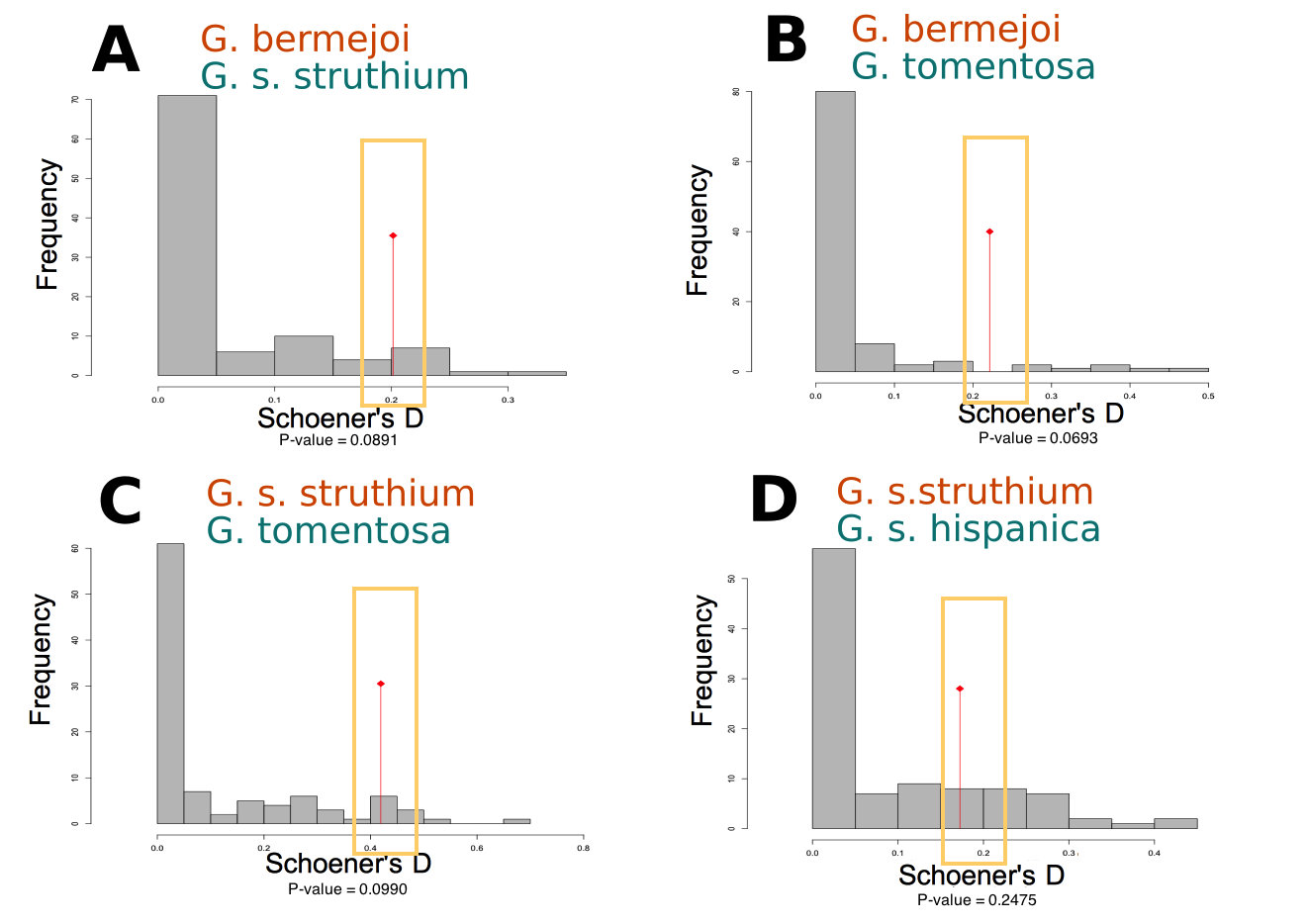

Supplement: S1 Fig — (TIF) [file pone.0206043.s005.tif]

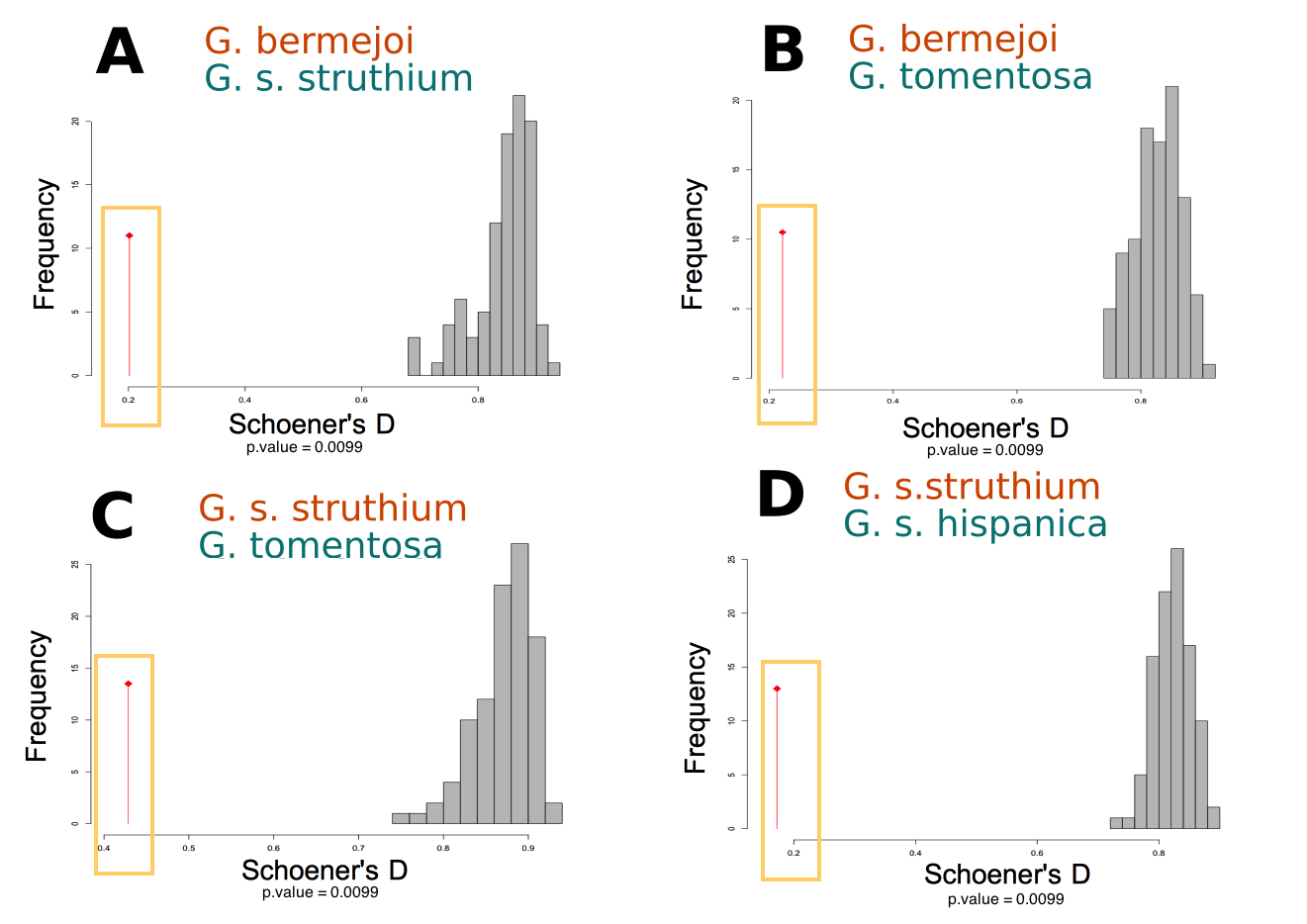

Supplement: S2 Fig — (TIF) [file pone.0206043.s006.tif]
